# 2,5-Dibromo­pyridine

**DOI:** 10.1107/S160053680900974X

**Published:** 2009-03-25

**Authors:** Rawhi H. Al-Far, Basem Fares Ali

**Affiliations:** a Faculty of Information Technology and Science, Al-Balqa’a Applied University, Salt, Jordan; b Department of Chemistry, Al al-Bayt University, Mafraq 25113, Jordan

## Abstract

In the title compound, C_5_H_3_Br_2_N, C—H⋯N hydrogen-bonding inter­actions and Br⋯Br inter­actions [3.9418 (3) and 3.8986 (3) Å] connect the mol­ecules into planar sheets stacked perpendicular to the *b* axis. In addition, pyrid­yl–pyridyl inter­sheet π–π stacking inter­actions [centroid–centroid distance = 4.12 (1) Å] result in a three-dimensional network.

## Related literature

For hydrogen bonding, see: Desiraju (1997 ▶). For related structures, see: Al-Far & Ali (2007 ▶, 2008 ▶); Ali & Al-Far (2008 ▶); Ali *et al.* (2008*a* ▶,*b* ▶). For bond-length data, see: Allen *et al.* (1987 ▶). For theoretical analysis, see: Awwadi *et al.* (2006 ▶, 2007 ▶).
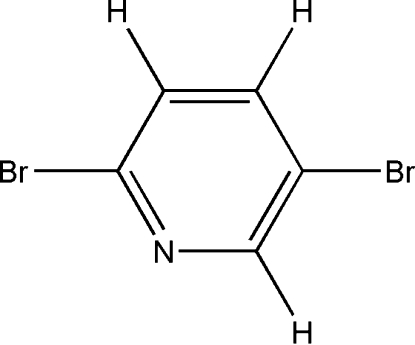


## Experimental

### 

#### Crystal data

C_5_H_3_Br_2_N
*M* *_r_* = 236.90
Orthorhombic, 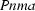

*a* = 6.1063 (4) Å
*b* = 6.5442 (4) Å
*c* = 15.8196 (9) Å
*V* = 632.17 (7) Å^3^
*Z* = 4
Mo *K*α radiation
μ = 12.71 mm^−1^
*T* = 90 K
0.46 × 0.21 × 0.14 mm

#### Data collection

Bruker SMART APEX diffractometer
Absorption correction: numerical (*SADABS*; Bruker, 2004 ▶) *T*_min_ = 0.053, *T*_max_ = 0.170
8997 measured reflections
996 independent reflections
887 reflections with *I* > 2σ(*I*)
*R*_int_ = 0.030

#### Refinement

*R*[*F* ^2^ > 2σ(*F* ^2^)] = 0.021
*wR*(*F* ^2^) = 0.054
*S* = 1.05
996 reflections
49 parameters
H-atom parameters constrained
Δρ_max_ = 0.68 e Å^−3^
Δρ_min_ = −0.77 e Å^−3^

### 

Data collection: *SMART* (Bruker, 2006 ▶); cell refinement: *SAINT-Plus* (Bruker, 2006 ▶); data reduction: *SAINT-Plus*; program(s) used to solve structure: *SHELXS97* (Sheldrick, 2008 ▶); program(s) used to refine structure: *SHELXTL* (Sheldrick, 2008 ▶); molecular graphics: *XP* (Bruker, 2004 ▶) and *SHELXTL*; software used to prepare material for publication: *XCIF* (Bruker, 2004 ▶) and *SHELXTL*.

## Supplementary Material

Crystal structure: contains datablocks I, global. DOI: 10.1107/S160053680900974X/pv2146sup1.cif

Structure factors: contains datablocks I. DOI: 10.1107/S160053680900974X/pv2146Isup2.hkl

Additional supplementary materials:  crystallographic information; 3D view; checkCIF report

## Figures and Tables

**Table 1:** Hydrogen-bond geometry (Å, °)

*D*—H⋯*A*	*D*—H	H⋯*A*	*D*⋯*A*	*D*—H⋯*A*
C4—H4*A*⋯N1^i^	0.95	2.38	3.323 (3)	175

Symmetry code: (i) 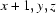.

**Fig. 1.**
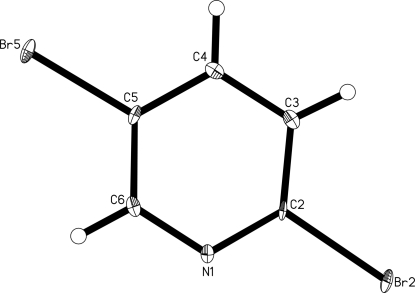
A molecular drawing of (I) shown with 50% probability ellipsoids.

**Fig. 2.**
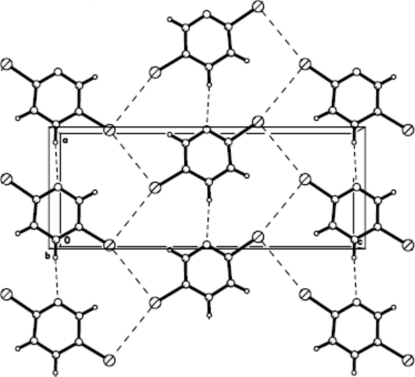
Packing diagram of (I) down the b-axis. Hydrogen bonding and Br···Br interactions are shown as dashed lines.

## supplementary crystallographic information

### Comment

Non-covalent interactions play an important role in organizing structural units in both natural and artificial systems (Desiraju, 1997). The interactions governing the crystal organization are expected to affect the packing and specific properties of solids. Intermolecular interactions are the essence of supramolecular chemistry, and the field of crystal supramolecularity seeks to understand intermolecular interactions by analyses of crystal packing. We are presently interested in the synthesis and the structural aspects of halo-metal anion salts containing different organic cations (Al-Far & Ali 2007, 2008; Ali *et al.* 2008*a, b*; Ali & Al-Far 2008). The title compound, (I), arose accidentally when attempting to crystallize CdBr_4_^2-^ with 2,5-bibromopyridinium cation. The compound had not been reported previously, thus, the structure of (I) has been characterized crystallographically and is presented here.

The bond distances and angles within the molecule of (I) (Fig. 1) are normal (Allen *et al.*, 1987). There is a non-classical hydrogen bonding interaction of the type C—H···N in the crystal structure which links molecules into one-dimensional chains (Fig. 2) parallel to the *a*-axis. The strength of the hydrogen bonds is represented by relatively short D···A distance and D—H···A angle (H···A = 2.38 Å, D—H···A 175°, Table 1). The resulting chains are further connected through Br···Br interactions in a zig zag arrangement to form sheets in the *ac* plane (Fig. 2); the Br···Br separation being 3.9418 (3) and 3.8986 (3) Å. The sheets are stacked along the *b*-axis with pyridyl···pyridyl π···π stacking intermolecular inreactions with distance between the centroids of the rings being 4.12 (1) Å. It is noteworthy that structural and theoritical results (Awwadi *et al.*, 2006; Awwadi *et al.*, 2007), show the significance of Br···Br bonding synthons in influencing structures of crystalline materials and in use as potential building blocks in crystal engineering *via* supramolecular synthesis.

### Experimental

The title compound crystallized during a reaction aiming to crystallize the anion [CuBr_4_]^2-^ with 2,5-dibromopyridinium cation. Colorless diamond like crystals of the title compound were obtained from an ethanolic solution of the reaction which involved a sequential addition to excess 2,5-dibrormopyridine (2.25 mmole) in ethanol of CdCl_2_ (1 mmole) and 60% HBr (1 ml) in ethanol.

### Refinement

Hydrogen atoms were positioned geometrically, with C—H = 0.95 Å, and constrained to ride on their parent atoms with *U*_iso_(H) = 1.2*U*_eq_(C).

### Crystal data

**Table:**

C_5_H_3_Br_2_N	*F*(000) = 440
*M**_r_* = 236.90	*D*_x_ = 2.489 Mg m^−^^3^
Orthorhombic, *P**n**m**a*	Mo *K*α radiation, λ = 0.71073 Å
Hall symbol: -P 2ac 2n	Cell parameters from 5485 reflections
*a* = 6.1063 (4) Å	θ = 2.6–30.0°
*b* = 6.5442 (4) Å	µ = 12.71 mm^−^^1^
*c* = 15.8196 (9) Å	*T* = 90 K
*V* = 632.17 (7) Å^3^	Diamond, colourless
*Z* = 4	0.46 × 0.21 × 0.14 mm

### Data collection

**Table:**

Bruker SMART APEX diffractometer	996 independent reflections
Radiation source: fine-focus sealed tube	887 reflections with *I* > 2σ(*I*)
graphite	*R*_int_ = 0.030
Detector resolution: 8.3 pixels mm^-1^	θ_max_ = 30.0°, θ_min_ = 3.6°
ω scans	*h* = −8→8
Absorption correction: numerical (*SADABS*; Bruker, 2004)	*k* = −9→9
*T*_min_ = 0.053, *T*_max_ = 0.170	*l* = −22→22
8997 measured reflections	

### Refinement

**Table:**

Refinement on *F*^2^	Primary atom site location: structure-invariant direct methods
Least-squares matrix: full	Secondary atom site location: difference Fourier map
*R*[*F*^2^ > 2σ(*F*^2^)] = 0.021	Hydrogen site location: inferred from neighbouring sites
*wR*(*F*^2^) = 0.054	H-atom parameters constrained
*S* = 1.05	*w* = 1/[σ^2^(*F*_o_^2^) + (0.0364*P*)^2^ + 0.1337*P*] where *P* = (*F*_o_^2^ + 2*F*_c_^2^)/3
996 reflections	(Δ/σ)_max_ < 0.001
49 parameters	Δρ_max_ = 0.68 e Å^−^^3^
0 restraints	Δρ_min_ = −0.77 e Å^−^^3^

### Special details

**Table:**

Geometry. All e.s.d.'s (except the e.s.d. in the dihedral angle between two l.s. planes) are estimated using the full covariance matrix. The cell e.s.d.'s are taken into account individually in the estimation of e.s.d.'s in distances, angles and torsion angles; correlations between e.s.d.'s in cell parameters are only used when they are defined by crystal symmetry. An approximate (isotropic) treatment of cell e.s.d.'s is used for estimating e.s.d.'s involving l.s. planes.
Refinement. Refinement of *F*^2^ against ALL reflections. The weighted *R*-factor *wR* and goodness of fit *S* are based on *F*^2^, conventional *R*-factors *R* are based on *F*, with *F* set to zero for negative *F*^2^. The threshold expression of *F*^2^ > σ(*F*^2^) is used only for calculating *R*-factors(gt) *etc*. and is not relevant to the choice of reflections for refinement. *R*-factors based on *F*^2^ are statistically about twice as large as those based on *F*, and *R*- factors based on ALL data will be even larger.

### Fractional atomic coordinates and isotropic or equivalent isotropic displacement parameters (Å^2^)

**Table:**

	*x*	*y*	*z*	*U*_iso_*/*U*_eq_	
Br2	0.41023 (4)	0.7500	0.171199 (12)	0.01101 (9)	
N1	0.4781 (3)	0.7500	−0.00105 (12)	0.0077 (4)	
Br5	0.98268 (4)	0.7500	−0.173378 (13)	0.01214 (9)	
C2	0.5860 (4)	0.7500	0.07141 (12)	0.0063 (4)	
C3	0.8124 (3)	0.7500	0.07962 (13)	0.0089 (4)	
H3A	0.8808	0.7500	0.1336	0.011*	
C4	0.9342 (4)	0.7500	0.00590 (14)	0.0088 (4)	
H4A	1.0897	0.7500	0.0077	0.011*	
C5	0.8236 (4)	0.7500	−0.07079 (12)	0.0074 (4)	
C6	0.5958 (4)	0.7500	−0.07196 (12)	0.0083 (4)	
H6A	0.5221	0.7500	−0.1248	0.010*	

### Atomic displacement parameters (Å^2^)

**Table:**

	*U*^11^	*U*^22^	*U*^33^	*U*^12^	*U*^13^	*U*^23^
Br2	0.01285 (15)	0.01709 (15)	0.00310 (12)	0.000	0.00345 (7)	0.000
N1	0.0081 (9)	0.0110 (9)	0.0041 (8)	0.000	0.0000 (6)	0.000
Br5	0.01335 (15)	0.01839 (15)	0.00466 (13)	0.000	0.00480 (7)	0.000
C2	0.0095 (11)	0.0078 (10)	0.0015 (8)	0.000	0.0012 (7)	0.000
C3	0.0087 (10)	0.0114 (10)	0.0066 (9)	0.000	−0.0024 (8)	0.000
C4	0.0067 (9)	0.0114 (10)	0.0084 (10)	0.000	−0.0018 (8)	0.000
C5	0.0088 (10)	0.0096 (10)	0.0040 (9)	0.000	0.0026 (7)	0.000
C6	0.0100 (11)	0.0100 (11)	0.0048 (9)	0.000	−0.0019 (7)	0.000

### Geometric parameters (Å, °)

**Table:**

Br2—C2	1.909 (2)	C3—H3A	0.9500
N1—C2	1.322 (3)	C4—C5	1.389 (3)
N1—C6	1.332 (3)	C4—H4A	0.9500
Br5—C5	1.891 (2)	C5—C6	1.391 (3)
C2—C3	1.389 (3)	C6—H6A	0.9500
C3—C4	1.383 (3)		
			
C2—N1—C6	117.43 (19)	C3—C4—H4A	120.8
N1—C2—C3	125.27 (19)	C5—C4—H4A	120.8
N1—C2—Br2	115.88 (16)	C4—C5—C6	119.87 (19)
C3—C2—Br2	118.85 (15)	C4—C5—Br5	119.98 (17)
C4—C3—C2	117.15 (19)	C6—C5—Br5	120.15 (16)
C4—C3—H3A	121.4	N1—C6—C5	121.91 (19)
C2—C3—H3A	121.4	N1—C6—H6A	119.0
C3—C4—C5	118.38 (19)	C5—C6—H6A	119.0
			
C6—N1—C2—C3	0.0	C3—C4—C5—C6	0.0
C6—N1—C2—Br2	180.0	C3—C4—C5—Br5	180.0
N1—C2—C3—C4	0.0	C2—N1—C6—C5	0.0
Br2—C2—C3—C4	180.0	C4—C5—C6—N1	0.0
C2—C3—C4—C5	0.0	Br5—C5—C6—N1	180.0

### Hydrogen-bond geometry (Å, °)

**Table:**

*D*—H···*A*	*D*—H	H···*A*	*D*···*A*	*D*—H···*A*
C4—H4A···N1^i^	0.95	2.38	3.323 (3)	175

Symmetry codes: (i) *x*+1, *y*, *z*.
